# The Dental Office and Laboratory

**Published:** 1887-02

**Authors:** 


THE DENTAL OFFICE AND LABORATORY.—This quarterly journal begins its third series with the January number and has changed its form to that of a pamphlet of some twenty-five pages, which presents a very pleasing appearance. It is now edited by Theodore Chupein, who has long been a contributor to its pages. Publishers: Johnson & Lund, Philadelphia.

